# Lipase from *Rhizopus oryzae* R1: in-depth characterization, immobilization, and evaluation in biodiesel production

**DOI:** 10.1186/s43141-020-00094-y

**Published:** 2021-01-05

**Authors:** Shimaa E. Helal, Hemmat M. Abdelhady, Khadiga A. Abou-Taleb, Mervat G. Hassan, Mahmoud M. Amer

**Affiliations:** 1 Department of Botany, Faculty of Science, Benha University, Benha, 13518 Egypt; 2 College of Life Science and Technology, Huazhong Agricultural University, Wuhan, 430070 China; 3 Department of Agricultural Microbiology, Faculty of Agriculture, Ain Shams University, Cairo, Egypt

**Keywords:** *Rhizopus oryzae*, Characterization, Lineweaver-Burk plot, Immobilization, Biodiesel production

## Abstract

**Background:**

Rhizopus species is among the most well-known lipase producers, and its enzyme is suitable for use in many industrial applications. Our research focuses on the production of lipase utilizing waste besides evaluating its applications.

**Results:**

An extracellular lipase was partially purified from the culture broth of *Rhizopus oryzae* R1 isolate to apparent homogeneity using ammonium sulfate precipitation followed by desalting via dialysis. The partially purified enzyme was non-specific lipase and the utmost activity was recorded at pH 6, 40 °C with high stability for 30 min. The constants *K*_m_ and *V*_max_, calculated from the Lineweaver-Burk plot, are 0.3 mg/mL and 208.3 U/mL, respectively. Monovalent metal ions such as Na^+^ (1 and 5 mM) and K^+^ (5 mM) were promoters of the lipase to enhance its activity with 110, 105.5, and 106.5%, respectively. Chitosan was used as a perfect support for immobilization via both adsorption and cross-linking in which the latter method attained immobilization efficiency of 99.1% and reusability of 12 cycles. The partially purified enzyme proved its ability in forming methyl oleate (biodiesel) through the esterification of oleic acid and transesterification of olive oil.

**Conclusion:**

The partially purified and immobilized lipase from *Rhizopus oryzae* R1 approved excellent efficiency, reusability, and a remarkable role in detergents and biodiesel production.

## Background

Lipases (triacylglycerol acylhydrolases E.C. 3.1.1.3) catalyze the breakdown of ester bonds of triglycerides at the interface between aqueous and oily layers [1]. Among different sources of lipases, those of microbial origin are mostly extracellular that are produced during the fermentation process. To separate lipase, cells are removed by either filtration or centrifugation then the purification strategies are applied to concentrate the cell-free culture. About 80% of these strategies use the precipitation technique with 60% of this percent involves the ammonium sulfate method [2, 3]. Specificity is a discriminative character of lipases and it can be categorized into positional, substrate, and stereo-specificity. Regarding the first category, lipases are subdivided into 1,3-positional-specific and non-positional-specific [4]. Hou and Shimada [5] reported that the positional specificity of lipases depends on their sources, e.g., most of the lipases produced by fungi are 1,3-positional-specific.

Employment of free lipases as industrial biocatalysts faces some problems, so immobilization is considered as an excellent solution for such barriers as it enhances the enzyme activity, selectivity, stability, and specificity. In addition, it improves lipase recycling and product purity that subsequently improves the process economy [6]. If compared to free enzymes, immobilized enzymes are more resistant and allow multiple reuses, and continuous operation of enzymatic processes [7]. A study by Pandey’s group [8] focused on the role of enzyme immobilization in improving the stability and subsequently increasing the reusability of enzymes.

The efficiency of the support for the success of the immobilized enzyme depends mainly on its surface area, porosity, and surface functionality [9]. In this respect, chitosan, which is a poly *N*-acetylglucosamine, identified as a perfect support because of its physical properties such as nontoxicity, biocompatibility, physiological inertness, biodegradability, high affinity to proteins, and antibacterial properties. Additionally, the existence of functional amino and hydroxyl groups makes it susceptible to chemical treatments [10]. One of the important types of immobilization methods is cross-linking in which the amino groups of the enzyme are connected covalently to the functional groups of the cross-linker such as glutaraldehyde to create a new bond for increasing the stability of the enzyme [11–13]. Using glutaraldehyde as a cross-linking agent increases the enzyme chance for successful reuse and gives it extraordinary advantages for a wide range of industries like food, fuel, and chemical as well as pharmaceuticals [14].

The utilization of lipases is shown to be effective than ordinary chemical syntheses in industrial applications such as pharmaceuticals, cosmetics, oleochemicals, detergents, and fragrances. This is beside their vital role in other applications like foods, biosensors, leather, paper pulp, and lipid-rich wastewater treatment [15]. Especially in the detergent industry, lipases are formulated with detergents to overcome using high temperature in the washing which consumes higher energy in addition to maintaining the quality of texture and fabrics [16].

Nowadays, the world is facing the problem of scarcity of fossil fuel, as it is a non-renewable source of energy. Excellent properties of biodiesel as a renewable source of energy and environment-friendly product paid a great attention to its production. Due to the high stability of enzymes as well as their convenient production, they are the best catalysts for producing biodiesel [17]. Biodiesel appears as an eco-friendly solution for a growing fossil fuel demand in the transport sector [18]. Furthermore, it is a clean-burning fuel, with lower emissions to the atmosphere of carcinogenic compounds and greenhouse gases, when compared to petrodiesel [19]. There is a great interest in the lipase use for the treatment of high lipid-content effluents to produce bioenergy [20].

## Methods

### Fungal strain and culture conditions for enzyme production

*Rhizopus oryzae* R1 was previously isolated from a soil sample collected from a gas station and identified using 18S rRNA as a potential lipolytic fungus (accession number: KJ417560) [21]. It was maintained on potato dextrose agar slant and kept at 4 °C until used. The modified fermentation medium formulated by Helal et al. [21] was carried out in 250 mL Erlenmeyer flasks containing 100 mL modified medium with the following composition (%): fish-frying oil, 2; peptone, 1.5; yeast extract, 1.5; and MgSO_4_.7 H_2_O, 0.04 and adjusted to pH 5.0. The flasks were sterilized in an autoclave at 121 °C for 20 min and cooled to room temperature (25 ± 2 °C). The flasks were inoculated with 8% v/v (10^8^ spores/mL) of tested strain and incubated at 28 °C on a rotary shaker (150 rpm) for 96 h. At the end of the incubation period, the fermented medium was filtrated using Whatman no. 1 filter paper, and then, lipase activity was assayed in the supernatant. Triplicates of all experiments were done to assure the results.

### Lipase assay

Colorimetric assay of lipase using the copper soap method as described by Veerapagu et al. [22]. Fatty acids liberated during hydrolysis of olive oil substrate by lipase can be determined colorimetrically using a cupric acetate-pyridine reagent. The reaction mixture consists of 1 mL of crude enzyme; 2.5 mL of olive oil was incubated for 5 min. Then, the reaction was stopped by adding 1.0 mL of 6 N HCl and 5 mL of benzene. The upper layer 4 mL was pipetted into a test tube and 1.0 mL of cupric acetate-pyridine reagent was added. The pyridine is used to adjust the pH at 6.0–6.2. The free fatty acids (FFA) dissolved in benzene yielding a blue color were determined by measuring the absorbance of benzene solution at 715 nm using spectrophotometer (AZZOTA SV110 Digital Visible Spectrophotometer New Jersey U.S.A). Lipase activity was determined by measuring the amount of FFA from the standard curve of oleic acid.

The amount of lipase that is required to release 1 μmole of fatty acid per minute is defined as one unit of enzyme activity and expressed as units per milliliter.

### Partial purification of lipase by ammonium sulfate fractionation

The crude culture filtrate (500 mL) was partially purified using different concentrations (0–80%) of solid ammonium sulfate under stirring conditions at 4 °C up to 24 h according to Ramadas and Ramadoss [23]. Then, it was centrifuged at 10.000 rpm for 10 min at 4 °C and the pellets of precipitated protein were collected. All the precipitate fractions (І–IV) were resuspended in a minimal amount of buffer (0.1 M phosphate buffer at pH = 7) and desalted by using Spectra/PorR, VWR 2003 dialysis membrane against the same buffer overnight at 4 °C with two times changes of the buffer. Total proteins and enzyme activity were determined after the dialysis [24].

### Determination of total protein

The measurement of protein was done as described by Hartree [25], using bovine serum albumin (BSA) as standard.

### Characterization of partial purified lipase

#### Determination of lipase positional specificity

The reaction mixture consists of 1 mL of purified enzyme and 2.5 mL of olive oil was incubated at 37 °C for 5 min under agitation 100 rpm. After the completion of the reaction, the hydrolysis products were extracted using *n*-hexane and separated into lipid classes. The lipid classes were analyzed on a thin layer chromatography (TLC) plate (5 × 9 cm) using the solvent system *n*-hexane to diethyl ether to acetic acid (80:20:1 by volume). To visualize the separated spots, the TLC plate was placed in an iodine chamber for about 1 min and the *R*_*f*_ values of lipid standards [26] were used to identify the product components.

#### Effect of substrate concentration

The effect of substrate concentrations on lipase activity was studied for determination of *V*_max_ and *K*_m_, the partially purified enzyme was incubated with different volumes of olive oil (0, 0.5, 1, 1.5, 2, 2.5, 3, and 3.5 mL which correspond to 0, 220, 330, 395, 440, 470, 494, and 512 mM, respectively) in the reaction mixture. The activity per unit time was determined under the standard assay conditions with each substrate concentration. The data obtained were plotted according to both Michaelis and Menten [27] and Lineweaver and Burk [28] to calculate *K*_m_ and *V*_max_ values as described by Maalej et al .[29].

#### Optimal pH and pH stability of lipase activity

The optimum pH was determined by measuring activity using optimum substrate concentration (440 mM which corresponds to 2.0 mL of olive oil) at different buffers (0.1 M) with various pH values: glycine-HCl buffer (pH 3.0), sodium acetate (pH 4.0 and 5.0), sodium phosphate (pH 6.0, 7.0, and 8.0), and sodium borate (pH 9.0 and 10.0). For studying pH stability, the enzyme solution incubated for 30, 60, and 90 min [30]. The percentage of relative lipase activity was calculated by comparing the activity of the treated enzyme with that of control (without buffer), which was considered 100%.

#### Optimal temperature and thermostability of lipase activity

Optimum temperature was assayed by measuring activity at optimum pH with varying temperature (20–90 °C), and the thermostability of the lipase enzyme was determined by incubating the partially purified enzyme solution at the temperatures range for 30–90 min. The relative lipase activity was measured according to the standard assay conditions. The activity of untreated enzyme (control) was taken as 100% [30].

#### Effect of chemical reagents [31]

The effect of chemical reagents on reaction velocity catalyzed by lipase was determined using the following:

- Metal ions: The enzyme activity was determined in the presence of different metal ions (Cu^++^, Mg^++^, Zn^++^, Ca^++^, Fe^++^, K^+^, and Na^+^) with varied concentrations of 1, 5, and 10 mM.
- Organic solvents: The influence of constant volume (2 mL) of different organic solvents such as acetone, benzene, methanol, chloroform, acetic acid, *n*-hexane, and ethanol on the lipase activity was studied. The substrate-solvent mixture was incubated at room temperature (25 ± 2 °C) for 1 and 2 h before measuring the enzyme assay.
- Chemical compounds: The enzyme activity was measured in the presence of various compounds such as ethylenediamine tetraacetic acid (EDTA), sodium dodecyl sulfate (SDS), tween 80 and Ariel (detergent) with 1 and 2%, and final concentration. Relative activities in the presence of each chemical were determined and compared with the control activity (hundred percent was assigned to the activity in the absence of these reagents).

### Enzyme immobilization

#### Formation of chitosan beads

Beads of chitosan were formed according to Nasratun et al. [6]. Typically, 3.0 g of chitosan powder was firstly dissolved in 100 mL of aqueous acetic acid (1%). The obtained solution was added drop by drop with the aid of a pipette into a coagulant bath that contains 100 mL of 1.0 M NaOH ethanolic solution (H_2_O: EtOH = 75: 25). Spherical beads were formed with diameters ranged between 1 and 2 mm. The mixture was left to settle overnight then the beads were filtered off, washed several times with deionized water and stored in it at 4 °C until use.

#### Characterization of chitosan beads

Two samples of chitosan beads (dried chitosan and chitosan with glutaraldehyde) were examined by using Fourier transform infrared spectrophotometer (FT-IR) spectra (Thermo Nicolet iS10 FT-IR Spectrophotometer). Freeze-dried samples were scanned in the range 4000–650 cm^−1^ at a resolution of 4.0 cm^−1^.

#### Immobilization methods

##### Method 1: Direct adsorption [6]

Eighteen grams of chitosan beads were soaked in *n*-hexane for 1 h under stirring (150 rpm). After removing hexane by filtration, 20 mL of the purified lipase was added to the support portion wise within 10 min under agitation condition (150 rpm). Stirring at room temperature (25 ± 2 °C) was continued for further 3 h followed by leaving under static conditions for 18 h at 4 °C, and then, the product was filtered off and rinsed with *n*-hexane.

##### Method 2: Adsorption-crosslinking [32]

Chitosan beads (18 g) were placed in phosphate buffer (pH 7.0) and stirred (150 rpm) at 30 °C with glutaraldehyde (3%) for 24 h. After that, the beads were filtered off, dried, and agitated (150 rpm) with 20 mL of purified enzyme for 3 h at room temperature (25 ± 2 °C). Phosphate buffer and distilled water were used to wash the activated beads from excess glutaraldehyde.

#### Reusability of the immobilized lipase [33]

The reusability of the immobilized lipase was investigated through using the beads for the hydrolysis reaction of olive oil as the substrate several times. After each run, the beads were separated from the reaction mixture, washed with distilled water, and stored at 4 °C until use in a next hydrolysis process. The first run was given an activity of 100% and other activities of other runs were calculated relative to this percent.

### Applications of lipase produced by *Rhizopus oryzae* R1

#### Washing performance

Application of partially purified lipase as a detergent additive was studied by using the literature method [34]. The oil-destaining efficiency of partially purified lipase was determined for its ability to clean fabrics from oil stains. Pieces (6 × 6 cm) of polycotton fabric were separately made dirty with two drops of waste frying oil and one drop of melted chocolate. After drying the fabric pieces, they were subjected to different oil-destaining treatments; plain water, lipase (2 mL) in water, pure lipase (2 mL), and detergent solution (0.1%). Each oil-stained fabric piece was placed in a 100 mL Erlenmeyer flask, soaked in the treatment solution, heated in water bath at 50 °C, and gently agitated for 30 min. After drying, the fabric pieces were observed for any oil stains residues.

#### Biodiesel production

##### Esterification [35]

An immobilized enzyme (2 g) was used as biocatalysts for the esterification of oleic acid (0.25 M) and methanol (0.4 M) in hexane. The reaction was carried out at 55 °C with shaking at 200 rpm for 16 h with heat-inactivated free enzyme (incubated at 75 °C for 1 h) as a control.

##### Transesterification

Utilization of oils for biodiesel production via enzymes was investigated by Yang et al. [36]; however, herein, we used the modified method of Yoo et al. [37]. Typically, to a mixture of olive oil (7.9 mL) and methanol (1.0 mL) in screw-capped glass tubes, 2.6 mL of purified lipase was added and incubated for 16 h at 40 °C with stirring at 200 rpm.

After the incubation period in both esterification and transesterification, samples (200 μL) were treated with 1.0 mL of *n-*hexane. Afterwards, TLC plates were used to separate the components of such samples. Methyl oleate (purity 99%) was spotted as reference biodiesel. The TLC plates were eluted with a mixture of *n*-hexane to ethyl acetate to acetic acid (90:10:1) and the developed spots were visualized by iodine vapors.

### Calculations

Specific activity (U/mg protein) = enzyme activity (U/mL)/protein content (mg/mL). Activity yield (%) = retained activity (U/mL)/initial activity (U/mL) × 100.

Enzyme purification (fold) = specific activity (U/mg protein)/initial specific activity (U/mg protein).

#### Michaelis-Menten equation [27]

*V* = *V*_max_ [S]/*K*_m_ + [S]; the equation is nonlinear where *V* = initial velocity of the reaction, *K*_m_ = Michaelis constant, *V*_max_ = maximum initial velocity, and [S] = substrate concentration.

#### Lineweaver-Burk plot [28]

A plot is generated form 1/V versus 1/[S] data. The linear equation used to determine the parameters:

$$ 1/\mathrm{V}=\left({\mathrm{K}}_{\mathrm{m}}/{\mathrm{V}}_{\mathrm{m}\mathrm{ax}}\right)\ \left(1/\left[\mathrm{S}\right]\right)+1/{\mathrm{V}}_{\mathrm{m}\mathrm{ax}}. $$

The equation of a straight-line *y* = mx + *b*. The slope of the line = *K*_m_/*V*_max_, and the intercept is 1/*V*_max_.

#### Immobilization efficiency (IE%)

It was calculated using the equation that is described by Talekar and Chavare [38].

$$ \mathrm{IE}\%=\left(\mathrm{activity}\ \mathrm{of}\ \mathrm{immobilized}\ \mathrm{enzyme}\ \left(\mathrm{IU}\right)/\mathrm{FU}-\mathrm{RUS}\right)\times 100 $$

where FU is the activity of free enzyme added and RUS is the activity of remaining enzyme in washed water and filtered solution.

#### Activity immobilization yield (AY%)

It was calculated using the following equation:

$$ \mathrm{AY}\%=\left(\mathrm{activity}\ \mathrm{of}\ \mathrm{immobilized}\ \mathrm{enzyme}\ \left(\mathrm{IU}\right)/\mathrm{activity}\ \mathrm{of}\ \mathrm{free}\ \mathrm{enzyme}\ \left(\mathrm{FU}\right)\right)\kern0.5em \times 100 $$

## Results

### Partial purification of lipase

The crude lipase produced by *Rhizopus oryzae* R1 was purified by ammonium sulfate fractionation followed by dialysis and the results are shown in Table 1. The maximum specific activity (1.56 U/mg, 87.5% yield) was recorded at fraction II 20-40% saturation with a 1.11-fold increase after purification.

**Table 1:** Partial purification of lipase from *R. oryzae* R1 by fractional precipitation of ammonium sulfate

Ammonium sulfate saturation %	Lipase activity (U/mL)	Protein content (mg/mL)	Specific activity (U/mg protein)	Yield (%)	Purification fold
**Crude extract**	210.0	150.0	1.40	100.0	1.00
**Fraction (I) 0–20%**	191.5	129.5	1.48	91.2	1.05
**Fraction (II) 20–40%**	183.8	118.3	1.56	87.5	1.11
**Fraction (III) 40–60%**	161.2	158.0	1.02	76.8	0.72
**Fraction (IV) 60–80%**	137.5	163.0	0.84	65.5	0.60

Specific activity (measures the enzyme’s purity) = (enzyme activity/protein content); yield (measures enzyme’s activity preservation) = (retained activity x 100/initial activity); and purification fold = (specific activity/initial specific activity)

### Characterization of the partial purified lipase

#### The positional specificity

**Fig. 1:**
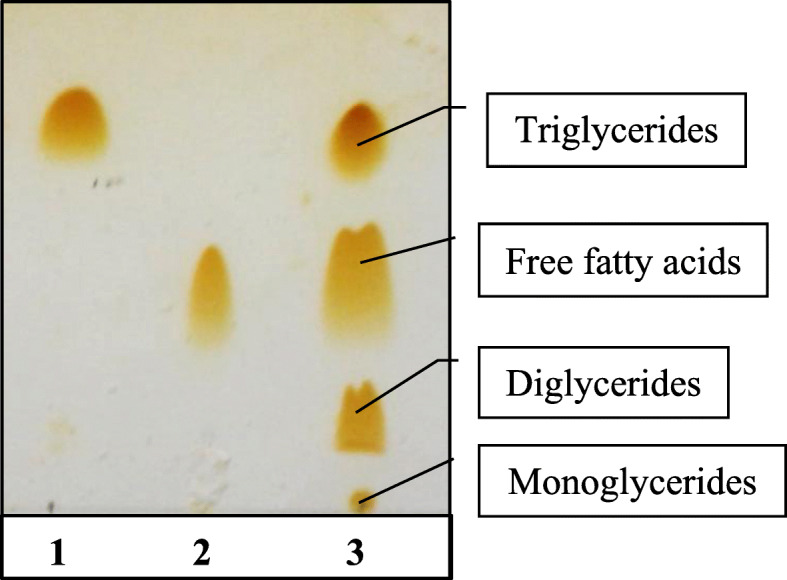
TLC plate for separation of lipid classes. Lane 1: Triglyceride standard. Lane 2: Oleic acid standard. Lane 3: Hydrolysis products of olive oil by *R. oryzae* R1 lipase

The positional specificity of lipase was investigated via determination of the hydrolysis products of olive oil through thin layer chromatography (TLC). Depending on the *R*_*f*_ values of lipid standards [26], the hydrolysis products were separated on the TLC plate (Fig. 1). Monoglycerides were observed near the origin of the TLC plate. Above monoglycerides, diglycerides (1,2- and 1,3-diglycerides) were found and the triglycerides were observed at the top of the plate, close to the solvent front. Free fatty acids were seen between diglycerides (DG) and triglycerides (TG).

#### Effect of substrate (oil) concentration

**Fig. 2:**
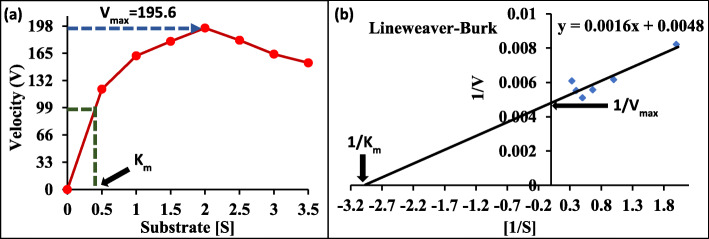
Kinetic plots based on the specific substrate for purified lipase produced by *Rhizopus oryzae* R1 using the Michaelis-Menten (**a**) and Lineweaver-Burk plot (**b**)

It is clear from Fig. 2 that the velocity of reaction (first-order reaction) increased with increasing substrate concentration until the point at which all active sites became filled. By this behavior, the lipase activity increased gradually with the substrate concentration up to 2 mL to give a maximum value of 195.6 U/mL with 108.2% relative activity to the control (2.5 mL of oil). At higher substrate concentration, there is great competition for the active sites, so the rate of reaction falls.

The *K*_*m*_ and *V*_max_ values determined using Michaelis-Menten plot and its derivative Lineweaver-Burk plot are 0.3 mg/mL and 208.3 U/mL, respectively.

#### Effect of pH and media stability

**Fig. 3:**
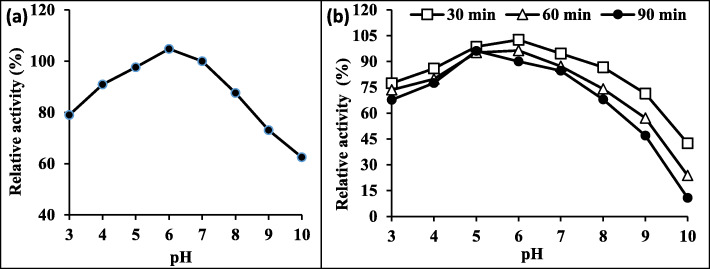
Effect of pH (**a**) and stability (**b**) on the activity of partially purified lipase produced by *R. oryzae* R1. *Initial activity (100%) = 195.6 U/mL at pH 7*

The enzyme characterization revealed that lipase produced by *Rhizopus oryzae* R1 was significantly active over a broad range of pH (3–10) in the presence of 2 mL of olive oil at 37 °C for 5 min. The maximum activity was attained at pH 6 with 104.8% relative activity then it decreased with either raising or lowering pH value (Fig. 3a).

Concerning the pH stability, the purified enzyme was stable for different periods (30–90 min) in various pH values and the highest stability was noticed after 30 min at pH 6 with 102.6% relative activity. In addition, it was observed that increasing the time leads to decreasing in lipase activity until it was lost at pH 8–10 being 10.8% as summarized in Fig. 3b.

#### Effect of temperature and thermostability

**Fig. 4:**
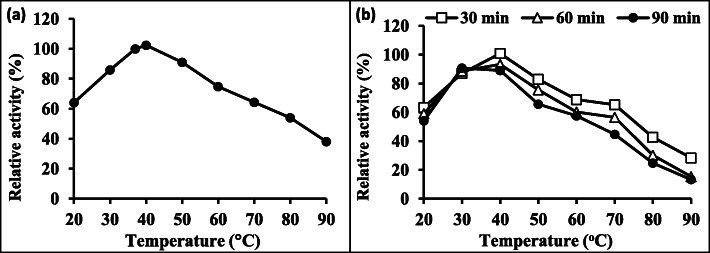
Effect of temperature (**a**) and stability (**b**) on the lipase activity produced by *R. oryzae* R1. *Initial activity (100%) = 205 U/mL at 37°C*

Temperature has a significant role on enzyme activity, so to determine the optimum temperature for lipase activity, the enzyme was tested at several temperatures (20–90 °C) using the preoptimized conditions (2 mL of olive oil and pH 6). The obtained results indicated that the enzyme reaction velocity increased with elevating temperature to 40 °C to reach the highest value (104.5%) and then it decreased (Fig. 4a).

The effect of temperature on enzyme reaction is usually given in terms of the temperature coefficient Q10, which is the factor by which the velocity is measured on raising the temperature by 10 °C [39]. The temperature coefficient of enzyme reactions usually lies between 1 and 2. Results showed that the Q10 values of enzyme were 1.34 and 1.19 between 20 and 30 °C and 30–40 °C.

Regarding the thermostability, the results clearly showed that the maximum stability (100.8% relative activity) was observed at 40 °C after 30 min. Amazingly, the enzyme retained about 83% of its original activity after incubation at 50 °C for half an hour; this demonstrates that the lipase produced by *R. oryzae* R1 can be used effectively with detergent in cleaning process at such temperature. On the contrary, the enzyme activity was mostly destroyed by heating at 90 °C giving 13.3% of relative activity and this may be due to the fracturing in the enzyme tertiary structure (Fig. 4b).

#### Effect of chemical reagents

**Fig. 5:**
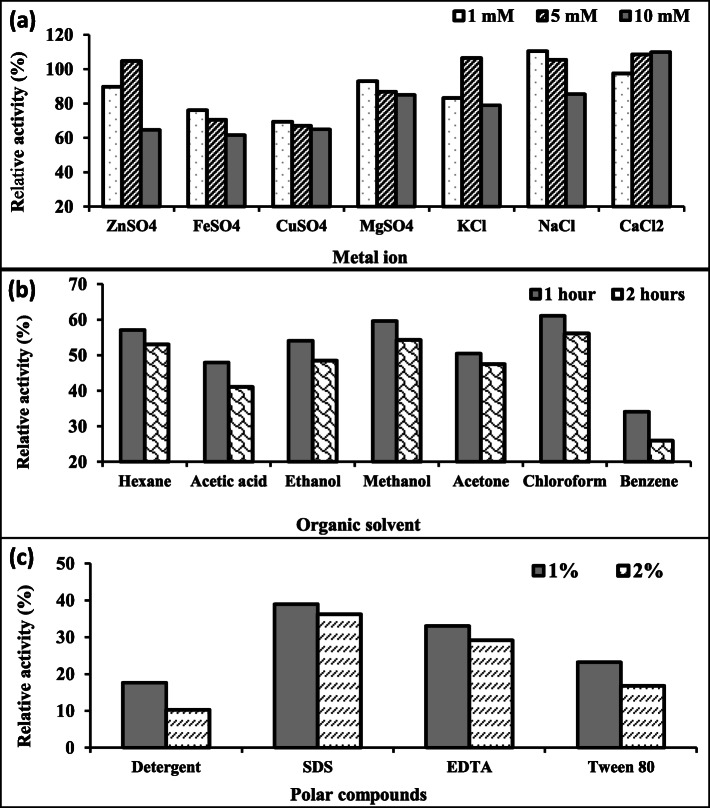
Effect of metal ions (**a**), organic solvents (**b**), and surfactant materials (**c**) on lipase produced by *R. oryzae* R1. *Initial activity (100%) = 210 U/mL*

Effect of various metal ions with varied concentrations (1, 5, and 10 mM) was studied on *R. oryzae* lipase. It was depicted that the monovalent metal ions (K^+^ and Na^+^) were promoters of the lipase, where lower concentrations of Na^+^ (1 and 5 mM) and K^+^ (5 mM) were more effective to enhance activity with 110, 105.5, and 106.5%, respectively. Some of the divalent metal ions (Fe^2+^, Cu^2+^, and Mg^2+^) reduced the activity by increasing the concentration up to 10 mM; however, the other divalent metal ions increased the lipase activity at 5 mM for Zn^2+^ and 5 and 10 mM for Ca^2+^ by 104.7, 108.6, and 110%, respectively (Fig. 5a).

With respect to the effect of organic solvents on the stability of lipase activity, the results illustrated in Fig. 5b indicated that all tested organic solvents have a negative effect on its stability. Furthermore, chloroform was found to have the lowest negative influence (61.1%) on the lipolytic activity while, benzene has the highest one (34.1%).

As for some chemical compounds, results showed that the enzyme could retain only up to 40% of its original activity. Higher concentrations of anionic surfactants (2%) such as detergent (Ariel) and SDS decreased the activity (being 10.3% and 36.2%, respectively) as represented in Fig. 5c and reported by Ebrahimpour et al. [40].

### Immobilization

#### Characterization of chitosan beads

**Fig. 6:**
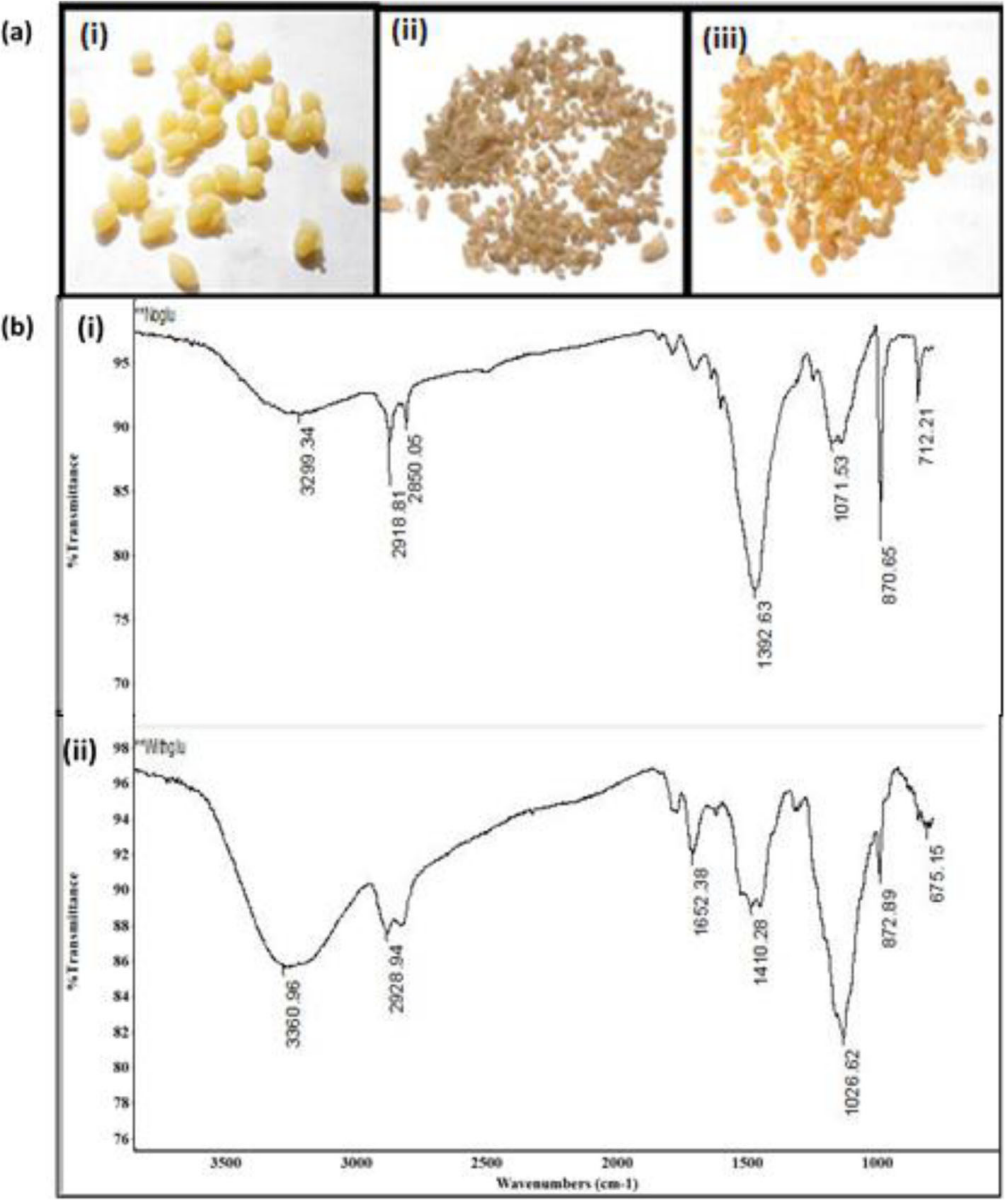
Characterization of chitosan beads. **a** Chitosan beads forms**: i** chitosan before drying, **ii** chitosan after drying, and **iii** chitosan with glutaraldehyde after drying. **b** FT-IR spectra of dried chitosan **(i)** and chitosan with glutaraldehyde **(ii)**

Different forms of chitosan beads are shown in Fig. 6a in which the dried form (Fig. 6aii) and chitosan with glutaraldehyde form (Fig. 6aiii) were used for immobilization. The FT-IR spectrum of both dried chitosan beads and chitosan with glutaraldehyde exhibited the absorption bands of the hydroxyl groups at 3299 and 3360 cm^-1^, a band of C–H stretching of the polymer at 2918 and 2928 cm^-1^ and (C–O) stretching at 1071 and 1026 (Fig. 6b). Moreover, an additional band at 1652 cm^-1^ was observed in dried chitosan with glutaraldehyde indicating the formation of imine (C=N) group which not found in dried chitosan beads (Fig. 6bii).

The results of FT-IR spectra strongly match those mentioned by Jiang et al. [41] who indicated the presence of stretching vibrations of hydroxyl peak at 3427 cm^−1^ and the occurrence of strong peaks at 2925 and 2855 cm^−1^ confirmed the existence C–H stretching vibration of the polymer backbone. Similarly, Nasra et al. [42] reported that the stretch vibrations of C–O are located at 1084 and 1032 cm^−1^. For cross-linked chitosan microspheres, the existing of an additional peak at 1656 cm^−1^, which clarifies stretching vibrations of the C=N bond, emphasizes the formation of Schiff’s base as a result of the reaction.

#### Immobilization of lipase on chitosan beads

In the present study, two methods were used to immobilize lipase and the results of the activity recovery and immobilization efficiency are given in Table 2. According to these results, the adsorption-crosslinking (method 2) was better than the direct adsorption (method 1) which gave the highest activity of the immobilized enzyme (192.7 U/mL**)** with 91.8% recovery and 99.1% efficiency. This might be due to the formation of weak bonds (mainly van der Waals, hydrogen bonds, and hydrophobic interactions) between chitosan and lipase. However, the adsorption-crosslinking method was highly efficient, because the presence of imine groups (C=N) that bind lipase covalently to chitosan beads as suggested by Liu et al. [43].

**Table 2:** Immobilization efficiency of lipase on chitosan beads

Immobilization method	FU (U/mL)	IU (U/mL)	RUS (U/mL)	AY (%)	IE (%)
Direct adsorption	210	175.4	29.3	83.5	97.1
Adsorption-crosslinking	210	192.7	15.5	91.8	99.1

*FU* free units, *IU* immobilized units, *RUS* retained units in solution, *AY* activity yield, *IE* immobilization efficiency = (IU)/(FU-RUS) × 100

#### Repeated batch operational stability of the immobilized lipase

**Fig. 7:**
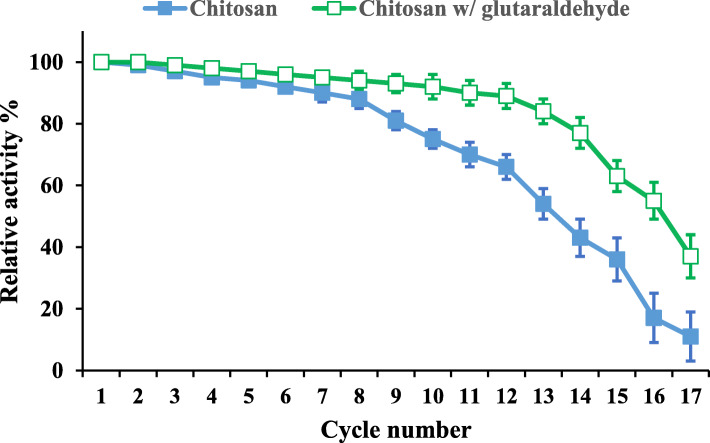
Reusability of immobilized lipase with direct adsorption on chitosan beads and adsorption-crosslinking on chitosan with glutaraldehyde. *Bars represent the standard deviations*

The most important of the immobilization is the reusability (enzyme recovery) in order to reduce losing the enzyme [44]. It is revealed from Fig. 7 that the immobilization of lipase by adsorption-crosslinking on chitosan with glutaraldehyde had a reusability of 12 cycles with 89% retained activity and be still applicable for the economical production. By direct adsorption on chitosan, the immobilized lipase can be reused for only 8 cycles; this might due to loss of enzyme from the surface of chitosan. From statistical analysis, it was found that the correlation coefficients between the activity of immobilized enzyme and cycle numbers are ranged from − 0.84 to − 0.94. So, it could be stated that the immobilized lipase activity by the adsorption-crosslinking was preferred in repetitive use than direct adsorption.

### Applications of purified lipase

#### Washing performance

**Fig. 8:**
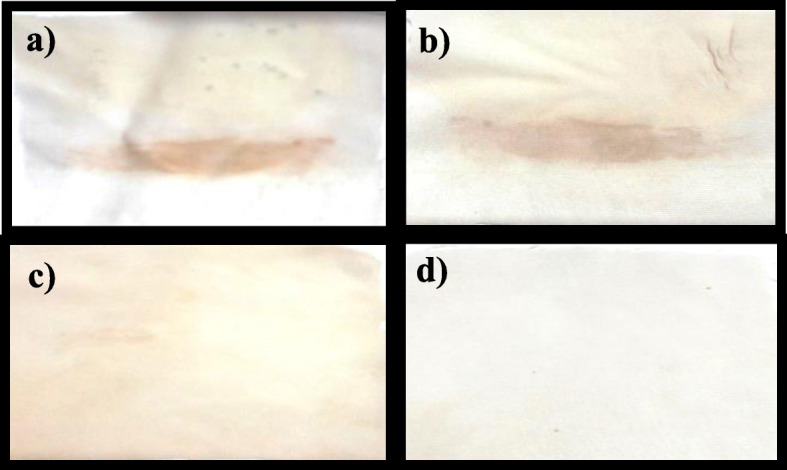
Wash performance of *R. oryzae* R1 lipase in combination with Ariel [detergent] at 50 °C for 30 min at 200 rpm. **a** Cloth stained with chocolate and oil, **b** stained cloth washed with water only, **c** stained cloth washed with enzyme only, and **d** stained cloth washed with detergent and enzyme

The wash performance of *R. oryzae* R1 lipase can remove a variety of stains especially oil ones from the cotton fabric surface at 50°C, and it is apparently clear that water had almost no effect on the stained cloth (Fig. 8a, b). Although the presence of the partially purified enzyme (as a sole cleaner) had a remarkable role in removing stains from cloth, the cleaning efficiency was not satisfactory (Fig. 8c). This may be due to the redepositing of the hydrolysis products (MG, DG, and FFA) on the fabric network beside the low emulsification power of the enzyme. Furthermore, Fig. 8d exhibited the highest cleaning effectiveness in the presence of lipase with the lowest concentration of detergent (0.1%) mixture.

#### Biodiesel production

**Fig. 9:**
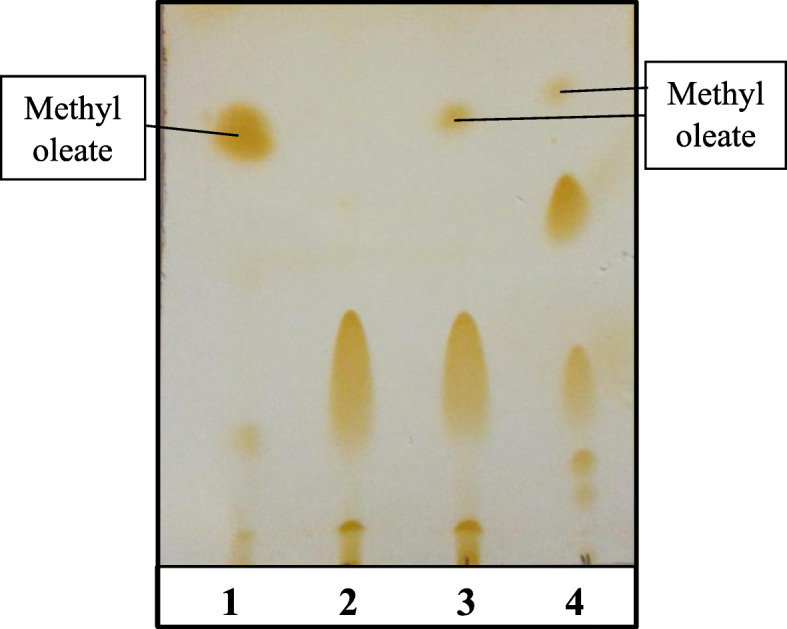
Production of biodiesel by lipase produced by *R. oryzae* R1 through esterification and transesterification using TLC plate. Lane 1: Standard methyl oleate. Lane 2: Standard oleic acid. Lane 3: Esterification products (immobilized enzyme with oleic acid). Lane 4: Transesterification products (purified enzyme with olive oil)

To evaluate the role of both immobilized and partially purified lipase in biodiesel production, TLC plate was used for detecting the formation of methyl esters of fatty acids (biodiesel). For both esterification and transesterification, spots for the hydrolysis products (monoglycerides, diglycerides, free fatty acids, and triglycerides) and methyl oleate, which indicate the ester formation, appeared clearly on the TLC plate (Fig. 9).

## Discussion

Lipase enzyme produced by *Rhizopus oryzae* R1 was purified to homogeneity, raising the concentration of ammonium sulfate to 60% and 80% led to a sharp drop in the purification fold. This is in proximity with Zouaoui and Bouziane [45] who indicated that the 40% saturation of ammonium sulfate afforded the highest specific activity of lipase produced by *Pseudomonas aeruginosa* with 60.89% recovery. On the other hand, Dey et al. [46] found that the *Pseudomonas* ADT3 lipase was partially purified about 2.9-fold over the crude extract with 64.4% recovery using 60–80% ammonium sulfate.

Regarding specificity, the *R. oryzae* R1 purified lipase is proved to be a random-specific enzyme (non-specific enzyme) utilizing TLC technique. Similarly, Rajan et al. [26] found that lipase produced by *A. fumigatus* is a non-specific enzyme. However, the lipase produced from *A. niger* was 1,3-specific as reported by Namboodiri and Chattopadhyaya [47]. The interconnection between the active site of enzyme and its specific substrate is a discriminative parameter of enzymatic reaction rate and subsequently enzyme activity. The lower value of *K*_*m*_ proves high affinity between enzyme and substrate, while *V*_max_ value assures high catalytic efficiency of lipase [48]. The values of *K*_m_ and *V*_max_ reported by Koblitz and Pastore [49] for the *Rhizopus* sp. lipase are 2.4 mmol/L and 277.8 U, respectively.

Enzymes are proteins, which are affected by changes in pH. High acidic or high alkaline pH values will lead to the ionization of amino acids atoms and molecules and change the shape and structure of proteins, thus damaging the function of proteins. Therefore, very high or very low pH will lead to the complete loss of the activity of most enzymes. The pH value at which the enzyme is most active is called the optimal pH value which was at pH 6. Higher temperatures tend to speed up the effect of enzyme activity, while lower temperatures decrease the rate of enzymatic reaction. At optimum temperature (40 °C), more molecules collide, increasing the chance that an enzyme will collide with its substrate. A study conducted by Zhou and others revealed that the purified lipase produced by *Aspergillus oryzae* had an optimal temperature at 40 °C [50]. Also, the enzyme from *Aspergillus flavus* was stable between 40 and 50 °C, with residual activity higher than 90% for 7 h [51].

Mono- and divalent alkali metal ions such as Li^+^, K^+^, and Ca^2+^ could increase the fungal lipase activity by 170% [52]; however, transition metal ions Fe^2+^ and Cu^2+^ cause a drop in the lipase activity by more than 40% as these ions can form complexes with the functional groups within the lipase-protein. The dramatic drop in the activity of *R. oryzae* R1 lipase by the action of solvents might be interpreted by that water-miscible organic solvents strip the water from enzyme, leading to the unfolding of the molecule with exposure of the inner hydrophobic residues and the denaturation occurred at a much faster rate than a pure aqueous system [53]. In addition, the drop in the activity in the presence of some chemicals may be due to the chelation of metal ions present with the enzyme by EDTA [34].

The yield of immobilized enzyme on chitosan/clay beads enhanced greatly and interpreted that by the presence cross-linking between NH_2_ group of chitosan and C=O of glutaraldehyde [54]. Moreover, glutaraldehyde can greatly increase the surface porosity of chitosan beads and the maximum capacity of immobilization [55]. As a result, immobilization process increases the stability for lipases in which the relative activity of the is retained after different reaction cycles [54]. In addition, the residual activity of immobilized lipase can withstand up to about 70% after 10 reaction cycles depending on the applied immobilization methods [56]. The cleaning efficiency of *R. oryzae* R1 lipase comes from its strong hydrolytic effect on triglycerides and exhibiting energy-efficient removal of oil stains from polycotton fabric [57]. Biodiesel production using lipases as catalysts is one of their major applications. In transesterification by *R. oryzae* R1 lipase, formation of methyl oleate is a two-step reaction consisting of hydrolysis and esterification of liberated fatty acids [58, 59]. However, in some cases, transesterification is one-step reaction proceeds via direct alcoholysis [60]. In addition, Rodrigues and coworkers carried out transesterification of jatropha oil with methanol, in a lipid/aqueous system by utilization of immobilized lipase/acyltransferase from *Candida parapsilosis* (CpLIP2) as a catalyst to produce biodiesel [61]. Utterly, the lipase produced by *R. oryzae* R1 can efficiently be used in various applications including cleaning process and biodiesel production.

## Conclusions

In the current research, the extracellular lipase produced by *Rhizopus oryzae* R1 was purified with ammonium sulfate fractionation followed by dialysis. Results showed that *Rhizopus oryzae* R1 lipase is a non-specific enzyme and exhibited high thermostability, excellent activity in a broad range of pH and temperature values, with the highest stability. Organic solvents and chemical substrates exhibited inhibitory behavior to the enzymatic activity. Glutaraldehyde-crosslinked chitosan was used as a perfect support for immobilization process. The purified and immobilized enzyme attained its efficiency in the detergent formulation and biodiesel production.

## Data Availability

Available on request.

## Abbreviations

TLC: Thin layer chromatography
BSA: Bovine serum albumin
FT-IR: Fourier transform infrared
EDTA: Ethylenediamine tetraacetic acid
SDS: Sodium dodecyl sulfate
FU: Free units
IU: Immobilized units
RUS: Retained units in solution
AY: Activity yield
IE: Immobilization efficiency
DG: Diglycerides
TG: Triglycerides
